# Black carbon yields highest nutrient and lowest arsenic release when using rice residuals in paddy soils

**DOI:** 10.1038/s41598-018-35414-3

**Published:** 2018-11-19

**Authors:** Jörg Schaller, Jiajia Wang, Md. Rafiqul Islam, Britta Planer-Friedrich

**Affiliations:** 1 Environmental Geochemistry, Bayreuth Center for Ecology and Environmental Research (BayCEER), University Bayreuth, Universitätsstraße 30, 95447 Bayreuth, Germany; 2 Department of Soil Science, Bangladesh Agricultural University, Mymensingh, 2202 Bangladesh

## Abstract

Rice straw increasingly remains on the fields for nutrient supply to the next generation of crop plants. It can be applied either fresh or after burning to black carbon or ash. A central concern during rice cultivation is accumulation of carcinogenic arsenic and the question arises how much rice straw application contributes to nutrient versus arsenic supply in paddy fields. Laboratory incubation experiments were performed to assess the effect of rice straw, black carbon and ash on element mobilization. Our experiments showed initially higher silicon and phosphorus release from black carbon compared to fresh straw amendments. However, more re-sorption to soil lead to finally slightly lower pore water concentrations for black carbon versus fresh straw amendments. Highest arsenic, iron, manganese and dissolved organic carbon concentrations were observed after fresh rice straw application. Black carbon and ash application lead to only minor increases of arsenic compared to controls without amendments. Overall, for silicon and phosphorus the soil acts as sink while for iron and arsenic it was the main source. In summary, burning of rice straw to black carbon prior to application seems to yield a high increase in desired nutrient and a decrease in undesired arsenic mobilization in paddy soils.

## Introduction

Rice is the major staple food worldwide. High yield production requires sufficient nutrient supply. Beside N for proteins, nucleic acids and chlorophyll, and P for forming important biomolecules (ATP, DNA)^1^, also silicon (Si) or trace elements such as iron (Fe) and sulfur (S) are important for good rice plant performance. As alternative to application of fertilizers during rice cultivation, rice straw may be used, because nutrients stored in the rice straw are then returned to the rice field soil after harvest. The most common way is to just leave the fresh rice straw in the field after harvesting and threshing for natural decomposition of the organic carbon and release of nutrients until the next crop cycle starts^2^. Typically, non-decomposed rice straw is still in the field when the next generation of plants is seeded or planted either because there is a short succession of crop cycles or the climate is too cold for high microbial activity and complete decomposition. Non-decomposed rice straw is then in most cases ploughed in before starting the next crop cycle and decomposition continues.

Some farmers also burn the rice straw to accelerate nutrient release^2^. Depending on burning temperature and completeness of mineralization, black carbon (~350 °C burning temperature), biochar (400 to 700 °C fixed-bed slow pyrolysis) or ash (full oxidation) forms. Numerous studies have shown the release of individual nutrients from either fresh straw (P, Si, sulfide^3–5^), black carbon (Si, P^6,7^), biochar (Si^8^), ash (Si, P), or a mixture of ash and charred material (Si, P, Fe, and As^9,10^). Rice straw is more prone to microbial decomposition compared to black carbon or ash, and hence induces a lower redox potential in the paddy soil pore water which promotes mobilization of Fe and P^11^. Where rice straw is burned by farmers after harvest directly on the soils by open field burning, fire conditions of common soil fires can be assumed^12^. The temperature of such fires is in the range of ~350 °C, but the maximum observed temperatures are up to 630 °C^13,14^. Such burning is fast and the availability of oxygen is high^13^, in most cases excluding the formation of biochar. During burning of straw to black carbon or ash, iron-oxides can be formed^15^ with subsequent sorption of elements like P. Rice does not only accumulate nutrients, but also toxicants. The most well-known toxicant in rice is arsenic (As), which is carcinogenic to humans^16^. Arsenic occurs ubiquitously in nature and is highly mobile under anoxic conditions, such as they exist in paddy soils. Three main species dominate in paddy soils: Arsenate under (micro)oxic conditions e.g. at the plant roots due to root radial oxygen loss, arsenite under anoxic conditions in the soil, and dimethylarsenate produced by methylating soil microorganisms^17^. All these As species can enter the rice plant due to their similarity to nutrients: Arsenite and dimethylarsenate enter the cell roots via aquaporin channels, which are also responsible for uptake of silicic acid^18,19^ and arsenate is taken up via phosphate transporters^20^. Arsenic levels in rice are therefore intrinsically linked to availability of nutrients. Under Si- and P-deficient conditions, As uptake increases. Oxidation of Fe(II) in soil solution to Fe(III) and its precipitation in the microoxic root rhizosphere with formation of Fe plaque decreases As uptake due to sorption^21^. Sulfur has also been reported to decrease As uptake^22^, though the exact mechanisms are not clearly understood. Hence, the concentration of those elements in soil pore waters affects As accumulation in rice plant tissues.

Accumulation of As in rice plants is not only a problem with respect to immediate grain consumption, but also for the practice to keep rice straw on the fields to retain the nutrients (and with them the toxicants). Concentrations of As in rice straw reach up to 100 mg kg^−1^ DW^−1^ ^23^. Concentrations in roots are comparably high, but quantitatively straw is more important for element cycling because of the significantly higher biomass. Currently available data show a significant As release from paddy soils after biochar application and as a result higher As accumulation in rice plants growing in those soils^24,25^. It was shown that microbes are able to use biochar for respiration^26–28^, which leads to enhanced mobilization of As due to dissolution of Fe minerals. However, typically, biochar is not produced by open field burnings practiced by farmers (see above), instead black carbon and ash are produced in different amounts depending on burning conditions^13,14^. However, despite knowledge about the effect of straw application and application of straw burned to a mixture of ash and charred material on element availabilities in paddy soils^9,10^, little is known about the different effects of black carbon and ash.

In our study, we investigated the release of the nutrients Si, P, Fe, S versus the toxicant As and other trace elements (Mn, Cu, Zn) under oxic versus anoxic conditions to simulate decomposition of straw. Furthermore, we tested the effect of straw decomposition on top of the soil versus straw ploughed into the soil. Our hypothesis was that application of fresh straw leads to a high mobilization of redox sensitive elements (P, Fe, As) and other elements accumulated in straw (e.g. Si). We expected less mobilization from black carbon or ash due to lower microbial activity (less labile carbon) leading to higher redox potential and formation of iron oxides as binding sites for e.g. P and As during burning. We compared nutrient versus arsenic release from different forms of rice straw (fresh straw, black carbon, ash) over time in incubations with and without paddy soil. Original release from straw was distinguished from secondary immobilization to soil and the major source (soil or straw) for each element was identified. In the end, we discuss in which form (fresh, black carbon, ash) rice straw is used best to maximize nutrient and minimize arsenic release.

## Results

### Effects on pH, redox potential, conductivity, DOC and element concentrations from incubation experiments with fresh straw under oxic and anoxic conditions in the absence of soil

**Figure 1:**
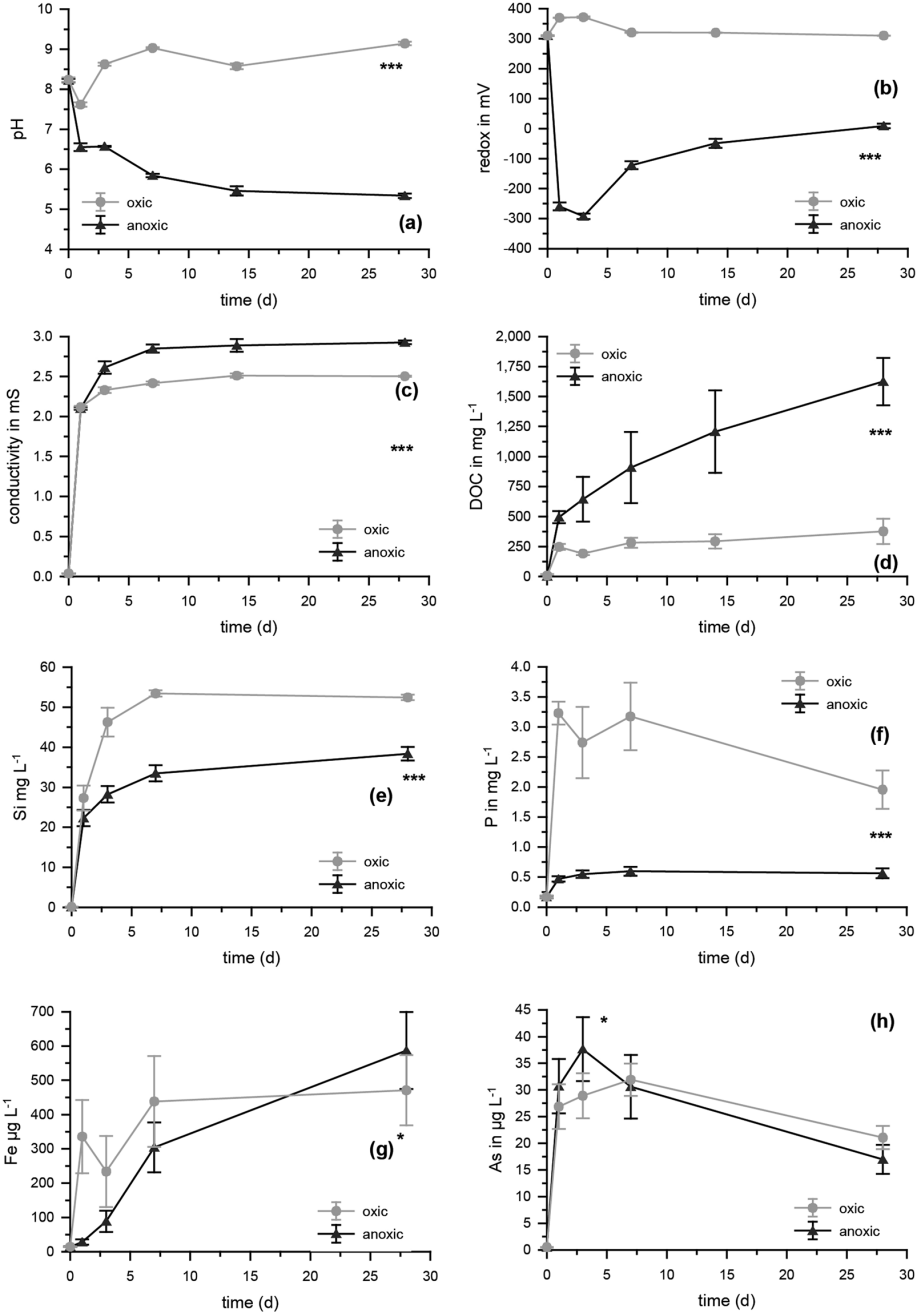
pH, redox potential, conductivity, and concentrations of DOC, Si, P, Fe, and As during decomposition of rice straw under oxic and anoxic conditions in experiments with straw and water in the absence of soil. Significant differences between the time series (*p* < 0.001, indicated as ***p < 0.01, indicated as **, and *p* < 0.05, indicated as *ANOVA) are shown, n = 5. For As, significant differences were found at 3 d between oxic and anoxic treatments (*p* < 0.05, *t*-test).

Decomposition of fresh rice straw proceeded significantly (*p* < 0.001, *t*-test) faster under oxic compared to anoxic conditions. After 4 weeks, 59 ± 3% of initial dry mass remained under oxic conditions, while it was 76 ± 2.3% under anoxic conditions. As expected, decomposition of fresh rice straw resulted in significantly (*p* < 0.001, df = 1, ANOVA) higher pH (F = 5089.800) and higher redox potential (F = 20558.055) under oxic conditions (pH 9.1 ± 0.1, E_H_ 300 mV) compared to anoxic conditions (pH 5.3 ± 0.1, E_H_ −250 mV) (Fig. 1a and b). Conductivity (F = 159.888) (Fig. 1c) and release of DOC (F = 463.831) (Fig. 1d) were lower under oxic (2.5 mS cm^−1^ and 376 ± 106 mg L^−1^) compared to anoxic conditions (2.9 mS cm^−1^ and 1,625 ± 197 mg L^−1^).

**Figure 2:**
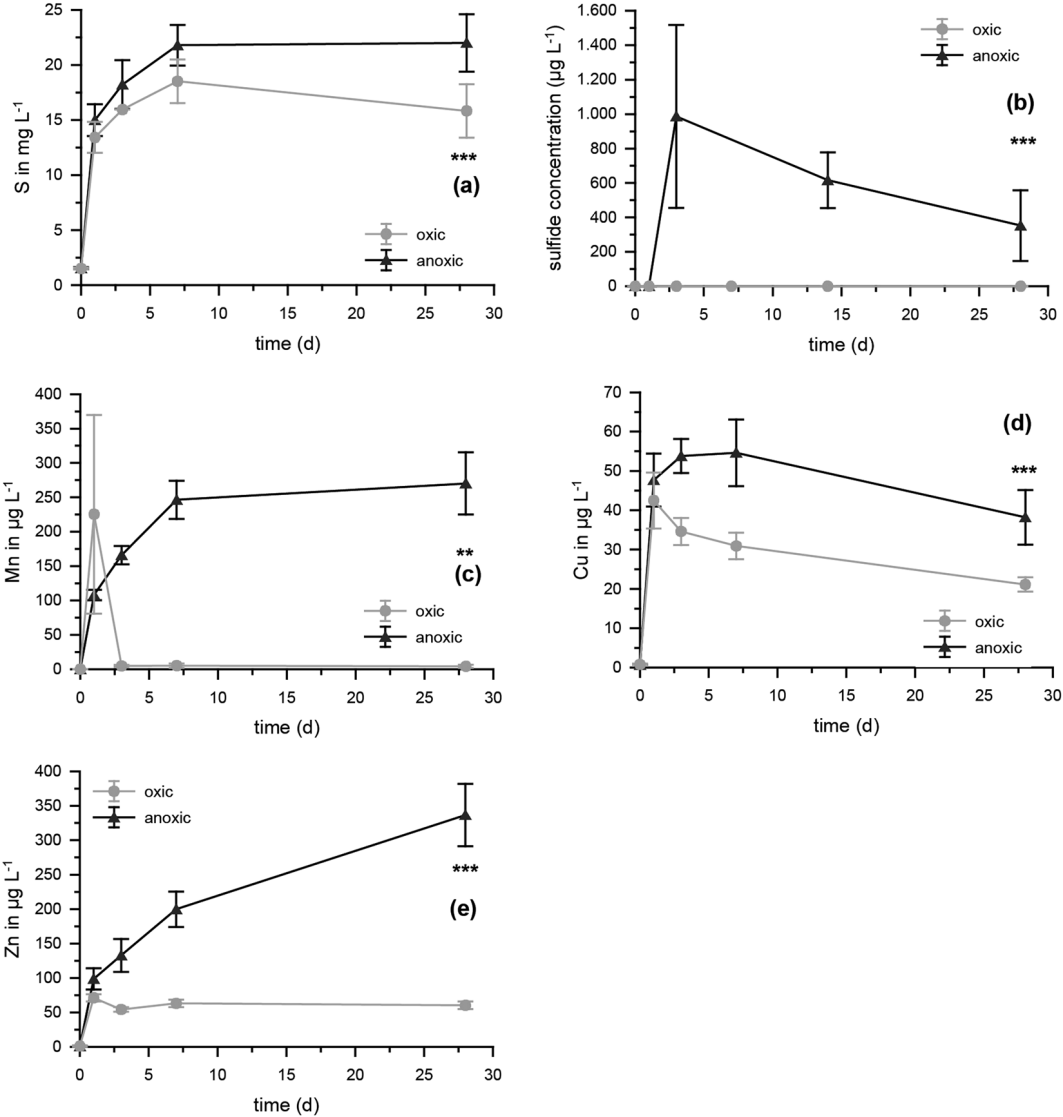
Mobilization of S, sulfide, Mn, Cu, and Zn during decomposition of rice straw under oxic and anoxic conditions in experiments with straw and water in the absence of soil. Significant differences between the time series (*p* < 0.001, ANOVA) are shown as ***n = 5.

The release of Si (F = 121.435) and P (F = 187.289) was significantly (*p* < 0.001, ANOVA) higher with the faster decomposition of fresh rice straw under oxic conditions (Fig. 1e,f). Overall, despite significant differences between oxic and anoxic treatments (Fig. 1g,h) (Fe, p < 0.05, F = 5.952 and As, p < 0.05, F = 5,652, both ANOVA), no clear pattern was observed for Fe and As. Total Fe release was initially slightly faster under oxic conditions, probably related to the faster decomposition. For As, the highest values were found at 3 d for anoxic treatments with 37.6 ± 6 µg L^−1^ and at 7 d for oxic treatments with 31.9 ± 3 µg L^−1^. Arsenic concentrations decreased to about half the maximum concentrations at the end of the experiment (28 d). Release of S (F = 20.851), Cu (F = 34.710), Zn (F = 127.652) (all p < 0.001, ANOVA), and Mn (p < 0.01, F = 9.514, ANOVA) was significantly lower under oxic compared to anoxic conditions. Under anoxic conditions, significant formation of sulfide (p < 0.001, F = 18.994, ANOVA) was observed (Fig. 2), with a maximum of almost 1 mg/L (5% of total S) after 3 days.

### Element mobilization from fresh straw, black carbon, and ash in the absence of soil

**Figure 3:**
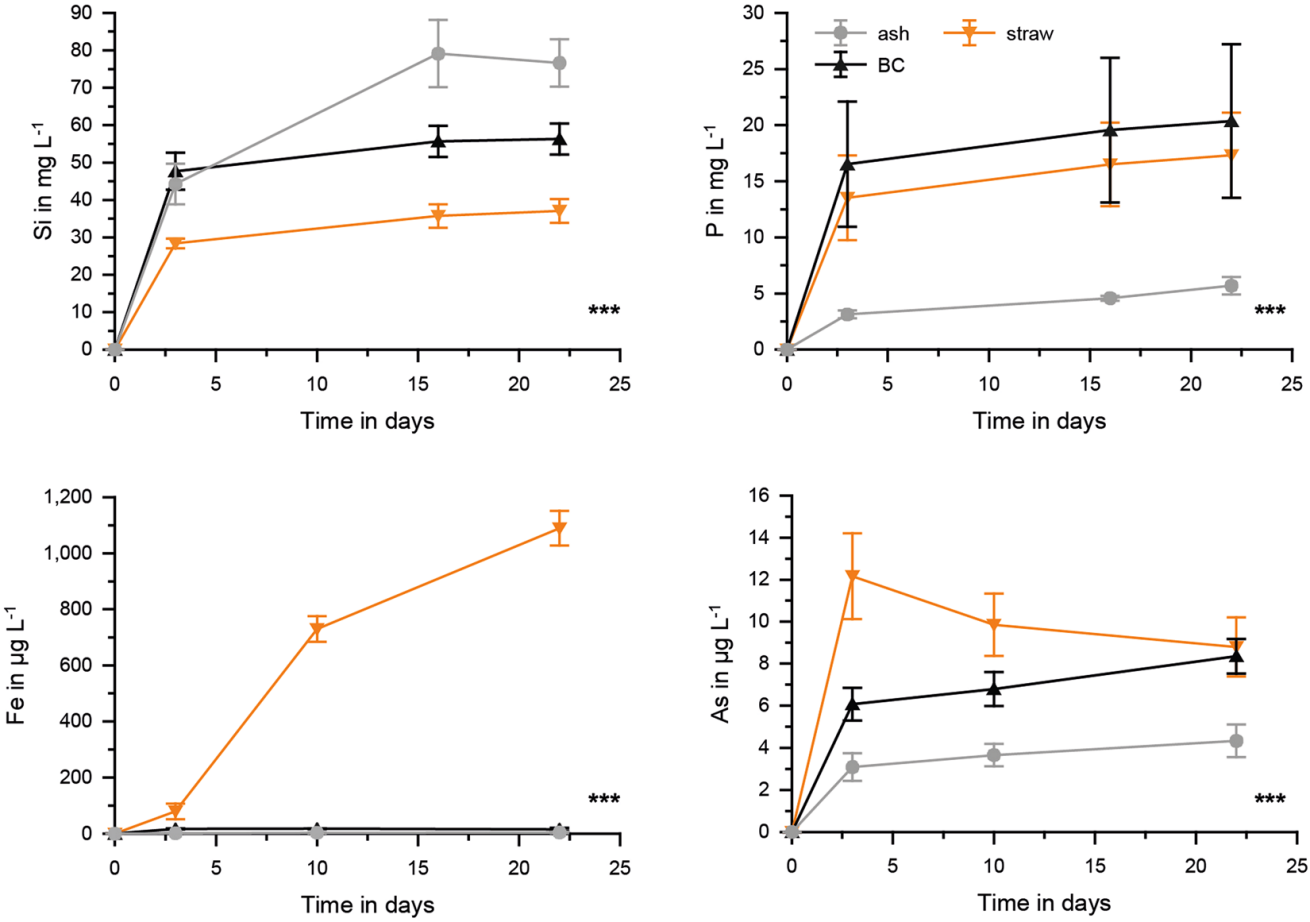
Concentration of Si, P, Fe and As in pore waters for the different applications (ash, black carbon (BC) and straw) without soils. Significant differences between the time series (*p* < 0.001, ANOVA) are shown as ***n = 4.

The chemical composition of rice straw, black carbon and ash used in the different experiments is listed in the supplementary material (Table A). Concentration of elements like P, Si, Fe, Cu, and Zn increases during burning from straw to black carbon to ash. The potentially volatile elements As and S showed no high losses to the atmosphere during burning. Instead, an enrichment was observed though to a slightly lesser degree compared to the other elements. In comparison to the experiments without soil and with high amounts of straw (30 g/800 mL tap water) (Fig. 1), the second set of experiments with only 5 g straw/500 mL tap water showed comparable mobilization of Si, higher mobilization of Fe and especially P (30 times higher), but three times lower mobilization of As. We found significant differences (*p* < 0.001, df = 2, ANOVA) between fresh rice straw, black carbon, and ash regarding Si (F = 23.004), P (F = 34.883), Fe (F = 17.624) and As (F = 45.360) mobilization. Comparing fresh rice straw, black carbon, and ash, the fresh rice straw released lowest Si, medium P and highest Fe and As concentrations in solution (Fig. 3). Black carbon and ash both released significantly (p < 0.005; Tukey post-hoc test) more Si, almost no Fe, and significantly less As compared to fresh rice straw. Phosphorous release was comparable between fresh straw and black carbon but significantly (p < 0.001; Tukey post-hoc test) lower for ash.

### Effects on pH, redox potential, conductivity, DOC and element concentrations from incubation of fresh straw, black carbon, and ash in the presence of soil

**Figure 4:**
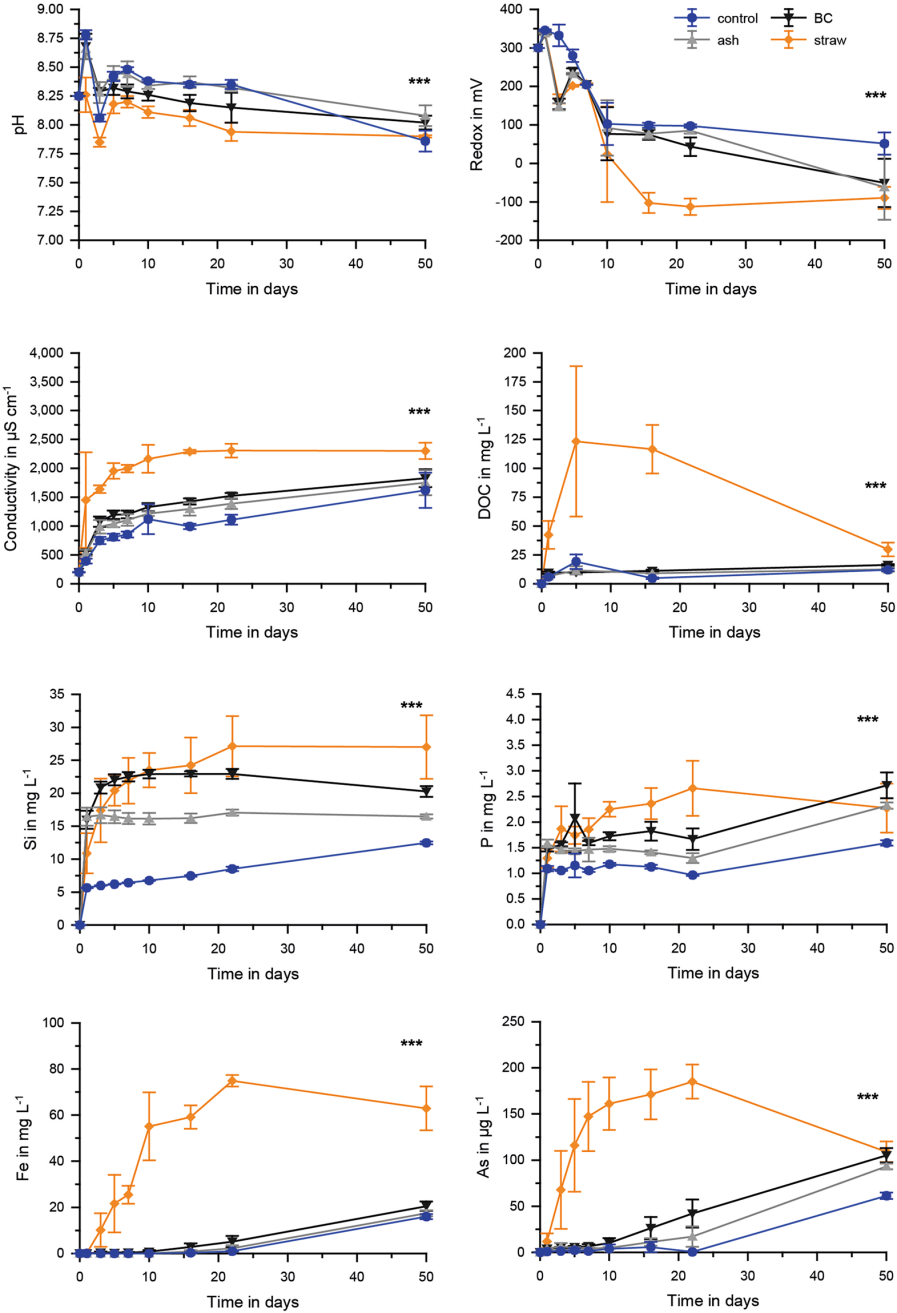
pH, redox potential, conductivity, concentrations of DOC, Si, P, Fe and As in pore waters for incubations of soil with different straw applications (fresh, black carbon (BC), ash) and the control treatment. Significant differences between the time series (*p* < 0.001, ANOVA) are shown as ***n = 5.

The chemical composition of soil used in the different experiments is listed in the supplementary material (Table A). Soil incubations with fresh rice straw were significantly (all *p* < 0.001, df = 3, ANOVA) different to those with black carbon, ash, or without any straw application (control) with regard to pH (F = 72.713), redox potential (F = 53.932), conductivity (F = 99.270) and DOC (F = 62.672) (Fig. 4). Fresh straw incubations had initially the lowest pH (7.8) and redox potential (−112 ± 20 mV) and the highest conductivity (2300 ± 150 µS cm^−1^) and DOC (123 ± 65 mg L^−1^). For comparison, controls without straw addition had pH 8.1 during the initial phase, redox potential 12 ± 45 mV, conductivity 1600 ± 300 µS cm^−1^, and DOC 20 mg L^−1^. Black carbon and ash treatments were comparable to each other with values for pH, redox potential, and conductivity between those for fresh straw addition and control while DOC was comparable to the control (n.s., Tukey post-hoc test).

**Figure 5:**
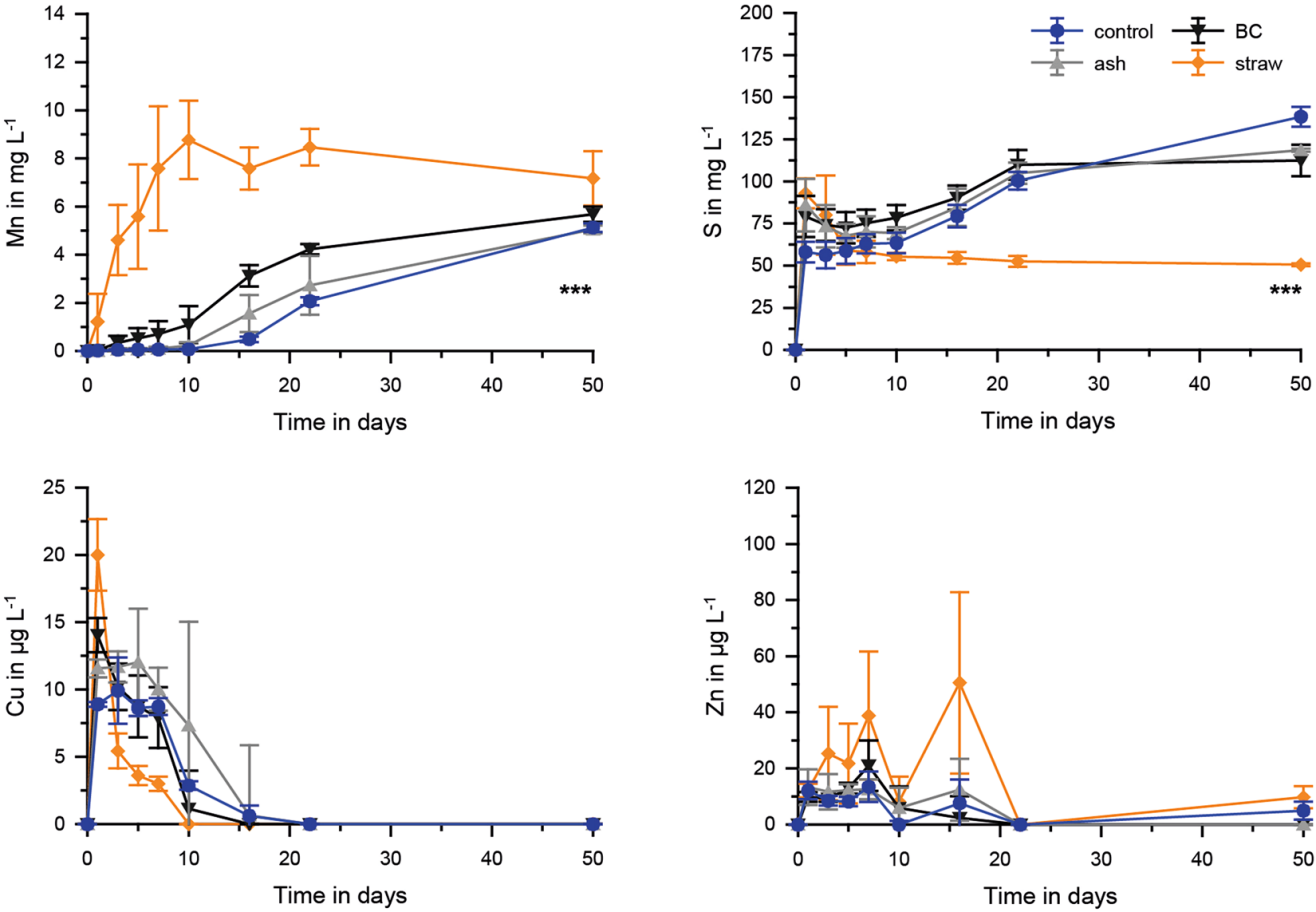
Mn, S, Cu and Zn concentration in pore waters for incubations of soil with different applications (ash, black carbon (BC) and straw) and the control treatment. Significant differences between the time series (*p* < 0.001, ANOVA) are shown as ***n = 5.

**Figure 6:**
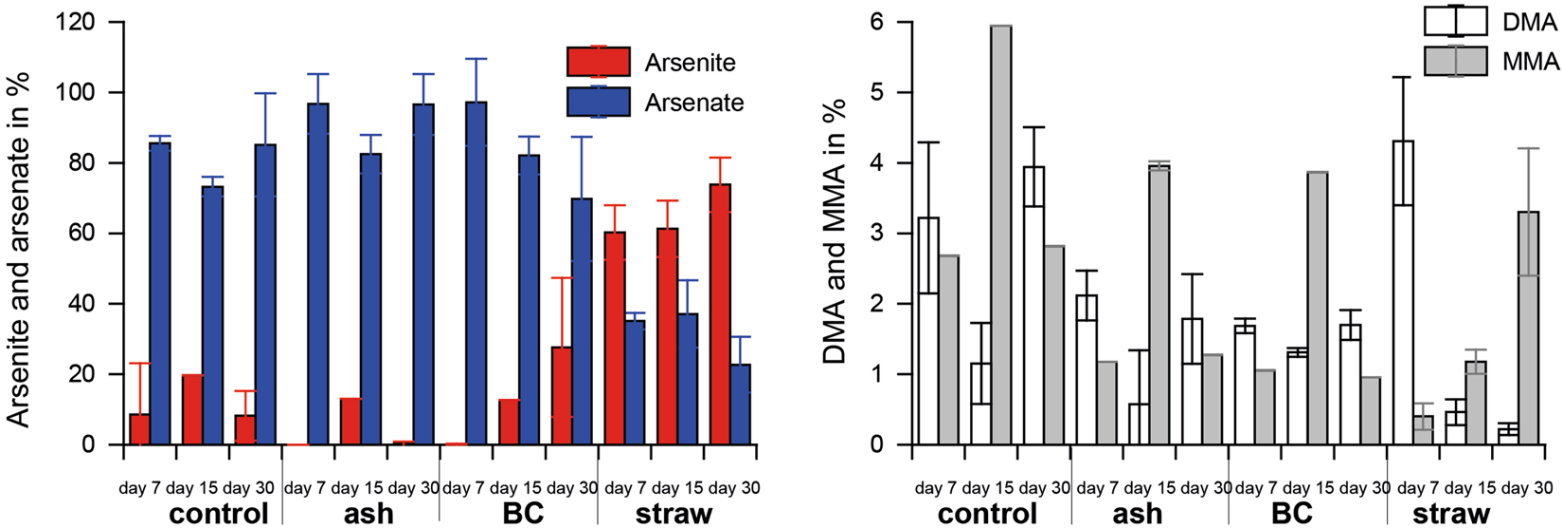
Concentration of the different As species in pore waters at selected days shown for arsenite, arsenate, DMA and MMA. BC is referring to black carbon application.

In the presence of soil, controls with no straw application showed the lowest Si, P, Fe, Mn, As and the highest S concentrations (p < 0.001; Tukey post-hoc test). Fresh rice straw showed higher Si and P values (Figs 3, 4) compared to all other treatments (p < 0.001; Tukey post-hoc test). For Fe and As (and similarly for Mn, Fig. 5) fresh straw application showed a much more rapid increase in concentrations compared to all other treatments and maximum concentrations were ~4 fold, ~2 fold, and ~2 fold, respectively, higher than those of the second highest mobilisation (black carbon application) (p < 0.001; Tukey post-hoc test). Arsenic speciation was not different between all treatments, except for those of straw application. For rice straw application the dominant species was arsenite, whereas for all other treatments arsenate was most dominant (Fig. 6). Arsenite (0–80%) and arsenate (20–100%) were by far the most dominant species. Monomethylarsenate (MMA) and dimethylarsenate (DMA) only contributed to minor percentages (<1–6% and <1–5%, respectively) and no further As peaks were detected during chromatographic separation. A temporal trend was only observed in the fresh straw applications with a decrease of DMA and an increase of MMA from day 7 to 15 and 30 (Fig. 6). Rice straw application showed lowest concentrations of S. Sulfide concentrations for all treatments were always below limit of detection (10 µg L^−1^). Black carbon and ash application were similar or slightly lower in Si and P concentrations compared to fresh straw, but Fe, As, Mn, and S concentrations were rather similar to the control treatment without any application (Fig. 5). For Cu and Zn, overall concentrations were low and no significant differences were found between the different treatments.

### Net element mobilization/immobilization

Referring element concentrations from the incubation experiments (with soil, Fig. 4) to concentrations from the experiments (without soil, Fig. 3) showed net ratios of <1 (immobilization in the presence of soil) for Si and P and ratios of >1 (additional mobilization in the presence of soil) for Fe and As (Table 1). Fresh straw application yielded the least net Si immobilization but also the highest net As mobilization in the presence of soil.

**Table 1:** Ratios of values at day 22 of experiments with soil divided by the values from samples without soil for the treatments with straw, black carbon or ash addition.

	Straw	Black carbon	Ash
Si	0.73	0.41	0.22
P	0.16	0.085	0.23
Fe	68	255	550
As	22	5	4

## Discussion

Rice straw application to paddy soils strongly increases nutrient availability for rice plants. Rice straw not ploughed into the soil will be decomposed under aquatic conditions (within the overlying water body) once the field is flooded for the next generation of rice plant cultivation. Our experiments showed that when rice straw is decomposed within the overlying water body under oxic conditions, decomposition proceeded much faster compared to anoxic conditions probably due to differences in microbial community and activity^29^ and much higher amounts of Si and P were mobilized under oxic conditions. The mobilization of Si from organic material during decomposition is generally slow^30^ and some Si fractions are still not dissolved after months, as shown before^31^. For P, maximum concentrations observed under oxic conditions in solution (3.2 mg L^−1^) were already close to complete mobilization from the straw we used (considering a total P concentration in the straw of ~116 µg P g^−1^ (Supplementary Material Table A) and the usage of 30 g straw per 800 mL experiment volume, maximum expectable P concentrations would be 4.3 mg L^−1^). Mobilization of the toxicant As was low under oxic conditions and most of the released As was re-bound to the remaining straw material, reducing the aqueous concentrations over time again (Fig. 1). Mobilization of As was comparably low under anoxic like under oxic conditions despite a known higher As mobility at low redox potentials. Considering a total As concentration in the straw of 1.1 ± 0.34 µg As g^−1^ (Supplementary Material Table A), maximum expectable total As concentrations would be 41 ± 12 µg L^−1^ As. Hence, the maximum concentrations observed (~38 µg L^−1^ As) were already close to complete mobilization from the straw even under oxic conditions. Overall low mobilisation of Fe (0.5 mg L^−1^ compared to a maximum expectable concentration of ~14 mg L^−1^) as well as decreasing As and P concentrations towards the end of our experiments may be potentially explained by binding to growing heterotrophic biofilm on the straw material, as previously shown for other organic material under decomposition^32,33^.

If rice straw material was burned to either black carbon or ash prior to application, mobilization of Si into paddy field surface water was much higher. These results confirm previous literature^6,8^. The lower availability of P from ash can potentially be explained by the high Ca content of rice straw^34^ leading to calcium phosphate formation during burning to ash strongly increasing the pH of the material^7^. The lower mobilization of As and Fe from the burning products (black carbon and ash) may be explained by formation of iron-oxides during burning^15^ with subsequent sorption of As.

Typically, however, in paddy soils fresh rice straw will not just remain on top of the field but be ploughed in and decompose in interaction with surrounding soil. Straw is clearly the source for Si and P (see results above and literature, e.g.^35^). However, the significantly lower pore water Si and P concentrations in experiments with soil compared to those without soils suggest that part of the released Si and P was adsorbed to the soil. Hence, paddy soils act as sink for pore water Si and P at each time point, representing snapshots of the adsorption-desorption equilibrium between soil solid and aqueous phase. The same effect was found for black carbon and ash application. For black carbon and ash, the immobilization of Si and P, by potential binding to soil minerals, was slightly higher compared to the straw treatment. In case of P, this higher immobilization in the black carbon and ash treatment may be attributed to the higher redox potential during incubation experiments probably due to changes in microbial community or lower microbial activity as a result of lower availability of labile carbon after burning and the potential higher amount of iron minerals formed during burning (see above). Compared to previous studies^9,10^, showing a negative effect of rice straw burning (leading to a mixture of ash and charred material) on P mobilization (after application in paddy soils), the data of the present study clearly shows that burning to black carbon leads to a much higher P mobilization compared to ash, which our data suggest for the first time when using rice straw as source material. Hence, burning conditions have a very strong influence on later nutrient availability, when using straw-based amendments, which our data suggest for the first time when using rice straw as source material. The strongly increased Fe mobilization in treatments with straw application can be explained by the lower redox potential mobilizing Fe from soil minerals. Under these conditions, also a strong increase of the toxicant As occurred, with As-speciation changing from dominantly arsenate to the even more toxic arsenite (Fig. 6). Microbial biofilm colonizing decomposing rice straw material generally has a high affinity for element binding^33^. Seemingly, however, As released from the soil due to lower redox potential as a result of straw decomposition is not re-adsorbed. Hence, application of rice straw leads to slightly enhanced nutrient (Si and P) availability but also to a strongly enhanced toxicant (As) availability.

High Si and P concentrations might have a further enhancing effect on As mobilization, because both compete with As for soil sorption sites^36–38^. The same elements will compete again for uptake by rice roots. The Si mobilization from especially straw and black carbon may help to reduce a possible Si deficiency at the end of the rice growing season^39^. In the presence of high Si availability derived from rice straw, black carbon or ash application, a decrease of As uptake may occur to a certain extent^18,19,40,41^. The same holds true for high P mobilization from the amendments potentially reducing the uptake of arsenate^20^. But there might be also an interdependency between Si and P, with Si strongly competing with P for soil binding sites^38^ controlling P availability for plants^42,43^.

In summary, our findings further emphasize difficulties with regard to use of rice residuals as fertilizers. Rice straw residuals are inevitably produced during rice cultivation. If they are not used on-site they have to be removed and additional synthesized fertilizer has to be applied. On the other hand, they are a naturally available and cheap source of fertilizer for rice farmers and in many countries they are still routinely burned to accelerate decomposition and nutrient availability for the next generation of plants. From a fertilization point of view, burning rice residuals to black carbon is the best option because it maximizes nutrient availability, while at the same timekeeping As mobilization low. Our results also suggest that, when burning, controlling fire conditions is crucial, because burning rice straw to ash, instead of black carbon, will result in much lower nutrient availability. Natural degradation of fresh straw on the other hand can only be recommended if As concentrations in the rice straw are really low. However, burning rice straw inevitably results in other environmental problems e.g. peaks in greenhouse gas emissions and increased atmospheric particle load. Sustainable future solutions will therefore need to balance both from an economical and from an environmental point of view costs and benefits of fresh rice straw use on-site (no transport costs and no peak atmospheric contamination, but highest arsenic mobility), burning on-site (no transport costs, highest nutrient availability, but peak atmospheric contamination), and removal with or without re-use after off-site treatment (e.g. controlled burning with no or little atmospheric contamination, but significant transport costs or costs for rice straw disposal).

## Methods

### Experimental setup

#### Experiments without soil

To separate mobilization from straw and immobilization to soil, we conducted incubation experiments without soil. In the first set of experiments, we used high amounts of fresh rice straw (from a rice field from Faridpur Sadar Upazila (23° 34′ 8.0″ N, 89° 46′ 58.6″ E; Bangladesh) cut to ~5 cm pieces, and air dried) (30 g to 800 mL tap water) in closed (but not gastight) 1 L glass bottles (Supplementary Material Table B). One subset of bottles was aerated by an aeration pump and air diffusers at a rate of 0.1 L min^−1^ to mimic fresh rice straw decomposition in the overlying water on top of the soil under oxic conditions. The other set of bottles was closed with a septum stopper to investigate the same fresh rice straw decomposition under anoxic conditions. This experiment was run in five replicates for 28 days. At the end of the experiment, the mass of the incubated straw was determined to estimate mass loss. In another set of experiments, we used the same rice straw material with a straw to water ratio of 5 g to 500 mL tap water and conducted experiments with fresh straw, black carbon, and ash (Supplementary Material Table B) for direct comparison to the soil incubations (see below). The same amount of straw was burned to either black carbon (at 350 °C until constant weight) or ash (at 550 °C until constant weight) using a muffle furnace according to Schaller, *et al*.^7^. These experiments were run in four replicates for 22 days.

### Experiments with soil

For each incubation experiment, 800 g soil (sieved to <2 mm) from paddy fields at the test site of Centre Français du Riz (Gimeaux, France) were transferred to a 1 L polyethylene bottle (Supplementary Material Table B). The bottles contained a rhizon sampler (Eijkelkamp, The Netherlands) attached to a valve at a soil depth of 7 cm horizontally. Four different treatments were done. One treatment was done with addition of 5 g rice straw (cut to ~5 cm pieces, air dried as mentioned above). The same amount of straw was then burned to either black carbon (at 350 °C until constant weight) or ash (at 550 °C until constant weight) using a muffle furnace according to Schaller, *et al*.^7^. The residues of the burnings were used as amendments and a control treatment was run without amendments. All amendments (straw, ash and black carbon) were well mixed with the soil using an end-over-end shaker for 2 hours, each with a replication of five. After that, 500 mL tap water was added to the soils, achieving a water layer of 4 cm above the soil and the bottles were closed (but not gastight), as in the experiment without soil (see above). The experiments were conducted at room temperature in the dark (all bottles were wrapped in aluminium foil entirely). For all treatments, the experiments were conducted under flooded conditions for 50 days. To investigate As speciation in further detail, we repeated the main experiment in a replication of three.

### Sampling, sample preparation and analysis

For the set of experiments with 30 g straw to 800 mL tap water, water samples were taken at the start and after 1 d, 3 d, 7 d, 14 d, and 28 d, filtered using 0.2 μm cellulose acetate filter (Rotilabo®) and stored in polyethylene vessels (which were confirmed by previous experiments to have no tendency to sorb the analyzed elements). For the experiments with 5 g straw to 500 mL tap water without soil, the sampling was done after 3, 16 and 22 days. Sample storage, digestion, and analyses were done as for the experiment with soil. For the experiments with soil, sampling was done after 1, 3, 5, 7, 10, 16, 22, and 50 days. Redox potential, pH, conductivity, and temperature were measured immediately (WTW pH meter pH 330 equipped with an WinLab Redox micro-electrode, a WinLab 423 combination pH electrode and an electrode for conductivity measurements (TetraCon 325), Mettler Toledo). Analysis of sulfide was done photometrically by the methylene blue method^44^.

Water samples for total element and DOC measurements were collected using sealed and evacuated glass vacuum bottles attached to the rhizon samplers by needles. Samples for total element measurements were stabilized with 250 µL 65% HNO_3_ and 150 µL 30% H_2_O_2_ to 10 mL water sample, in accordance with DIN-EN-ISO-5667^45^). Water samples for measurement of dissolved organic carbon (DOC, 7 mL) were frozen until analysis. Element analysis (except for Si) for water samples was done using inductively coupled plasma-mass spectrometry (ICP-MS, XSeries2, Thermo-Fisher). Rhodium was used as internal standard and re-analyses of a mid-concentration range calibration standard were used for internal drift correction control by checking a mid-range standard every 20 samples. Each element was calibrated using commercial ICP-MS single-element standards Calibration functions were recorded from mixed calibration samples, which were prepared from single element solutions (Bernd Kraft, Duisburg, Germany). Silicon was measured by inductively coupled plasma optical emission spectrometry (ICP-OES, Varian, Vista-Pro radial). Dissolved organic carbon was measured using a thermo-catalytic oxidation on a TOC-VCPN Analyzer (Shimadzu, Kyoto, Japan).

The element composition of the rice straw material and the soil was determined before the start of the experiments. Rice straw material was dried at 50 °C to constant weight and ground afterwards. A CEM Mars5 microwave digestion system (CEM Corporation, Matthews, NC, USA) was used to digest the ground rice straw (particle size <0.5 mm) in 3 mL of HNO_3_ and 2 mL H_2_O_2_^46^. For every set of samples digested, we used one blank. Chemical blanks and standard reference material NCS 73349 digested the same way like the samples were used to confirm accuracy of analysis (recovery 97%). The solution was analyzed for element content by ICP-MS (except of Si). Silicon was extracted from rice straw material by an alkaline digestion where 30 mg ± 1 mg of each sample were weighed into extraction vials, filled up with 30 mL 1% Na_2_CO_3_ and treated 5 h at 85 °C in a heating block. Samples were filtered through 0.2 µm cellulose acetate filters and stored at room temperature until analysis with inductively coupled plasma optical emission spectrometry (ICP-OES). Soil samples were digested in a microwave (see above) using an *aqua regia* extract. Element concentrations were determined by ICP-MS. To determine dissolved and exchangeable Si from soils we used the sodium acetate extraction according to Sauer, *et al*.^47^, because this method was shown to correlate with Si uptake by rice^48^. In short, 5 g of soil were incubated with 50 mL sodium acetate and adjusted to pH 4 for 5 h at 40 °C. The extracts were filtered (0.2 µm cellulose acetate) and measured by inductively coupled plasma optical emission spectrometry (ICP-OES). Carbon and nitrogen contents in the solid samples were measured using Elementar Vario El III (Hanau, Germany) in accordance with DIN-ISO-10694^49^.

For the experiment focusing on As speciation, sampling was done on sampling days with the expected highest As concentrations, based on the results of our main experiment with soil (sampling was done after 7, 15, and 30 days). Pore waters were collected again using rhizon pore water samplers (Eijkelkamp, The Netherlands) connected to sealed and evacuated glass vacuum bottles attached to the rhizon samplers by needles. Samples (2 mL) were stabilized with an iron-complexing agent (10 mmol L^−1^ diethylenetriaminepentaacetic acid DTPA), immediately flash-frozen (using dry ice), and stored (in 2 mL polyethylene vials) at −20 °C until analysis. To confirm that DTPA does not change As speciation, we did an immediate measurement of selected sample aliquots without any stabilization and confirmed match of results between stabilized and non-stabilized sample. Arsenic speciation was analyzed by anion exchange chromatography (Dionex ICS-3000 SP using an AG16/AS16 Ion Pac column, 4 mm, Dionex) coupled to ICP-MS in a slight adaptation of a method of Wallschläger and London^50^ using 10 mmol L^−1^ NaOH as starting eluent instead of 2.5 mmol L^−1^ in the original publication. Retention time of the species were verified by single standards. The peaks were well resolved. Sum of species was about 80% of total As and besides arsenite, arsenate, MMA, and DMA no additional As peaks were detected.

### Statistical analysis

Analysis of variance (ANOVA) was used to compare element water concentration of the different treatments, combined with a Tukey post-hoc test, and t-test was used to compare mass loss data for the experiment with only straw and without soil using SPSS version 21.0.

## Electronic supplementary material

Supplementary Information

## Data Availability

Data supporting the findings of the current study are available from the corresponding author on reasonable request. All data analyzed during this study are included in this published article.
