# Supplementary material for: Black carbon yields highest nutrient and lowest arsenic release when using rice residuals in paddy soils
Source: Sci Rep. 2018 Nov 19;8:17004. doi: 10.1038/s41598-018-35414-3 (PMC6242850; doi:10.1038/s41598-018-35414-3)
Supplement: Supplementary file 1 — Supplementary Information [file 41598_2018_35414_MOESM1_ESM.doc]

**Supplementary Material** to:

**Black carbon yields highest nutrient and lowest arsenic release when using rice residuals in paddy soils**

Jörg Schaller^1*^, Jiajia Wang^1^, Md. Rafiqul Islam^2^ and Britta Planer-Friedrich^1^

^1^ Environmental Geochemistry, Bayreuth Center for Ecology and Environmental Research (BayCEER), University Bayreuth, Universitätsstraße 30, 95447 Bayreuth, Germany

^2^ Department of Soil Science, Bangladesh Agricultural University, Mymensingh-2202, Bangladesh

^*^Corresponding author: Tel.: +49 921 55 3991, Fax: +49 921 55 2334; E-mail address: Joerg.Schaller@uni-bayreuth.de

Burning leads to a mass loss of ~72 % for black carbon and of ~87 % for ash. The chemical compositions of rice straw, black carbon, ash, and soil used in the different experiments are listed in Table A.

Table A: Total element concentrations (except for Si) of rice straw (triplicates, mean±SD) and soil element concentration (homogenized soil). Please note that Si (dissolved plus exchangeable) was determined after extraction.

| Element | Rice straw | BC | Ash | Soil |
| --- | --- | --- | --- | --- |
| C | 43±1.6% | 15±0.2% | 0.5±0.1% | 3.7±0.1% |
| N | 1.7±0.1% | 1.0±0.1% | 0.1±0.0% | 0.2±0.1% |
| P | 116±41 mg kg^-1^ DW^-1^ | 530±16 mg kg^-1^ DW^-1^ | 1,210±270 mg kg^-1^ DW^-1^ | 203 mg kg^-1^ DW^-1^ |
| Si | 32.4±0.4 g kg^-1^ DW^-1^ | 129±6 g kg^-1^ DW^-1^ | 298±12 g kg^-1^ DW^-1^ | 64.6 mg kg^-1^ DW^-1^ |
| Mn | 124±25 mg kg^-1^ DW^-1^ | 870±85 mg kg^-1^ DW^-1^ | 370±80 mg kg^-1^ DW^-1^ | 378 mg kg^-1^ DW^-1^ |
| Fe | 370±80 mg kg^-1^ DW^-1^ | 1,760±670 mg kg^-1^ DW^-1^ | 2,650±340 mg kg^-1^ DW^-1^ | 25 g kg^-1^ DW^-1^ |
| S | 1,700±460 mg kg^-1^ DW^-1^ | 6,970±230 mg kg^-1^ DW^-1^ | 8,060±1300 mg kg^-1^ DW^-1^ | 4.85 g kg^-1^ DW^-1^ |
| Cu | 5.9±1.6 mg kg^-1^ DW^-1^ | 28±06 mg kg^-1^ DW^-1^ | 42±7 mg kg^-1^ DW^-1^ | 28 mg kg^-1^ DW^-1^ |
| Zn | 25.3±3.4 mg kg^-1^ DW^-1^ | 80±6 mg kg^-1^ DW^-1^ | 155±28 mg kg^-1^ DW^-1^ | 88 mg kg^-1^ DW^-1^ |
| As | 1.1±0.34 mg kg^-1^ DW^-1^ | 5.4±1.2 mg kg^-1^ DW^-1^ | 9.4±1.5 mg kg^-1^ DW^-1^ | 9.49 mg kg^-1^ DW^-1^ |

Table B: Overview on the different experiments conducted.

| **Experiments (setup)** | **Treatments** | **Sampling days** | **Figures** |
| --- | --- | --- | --- |
| 30 g straw in 800 mL water **without soil** | oxic or anoxic | 1, 3, 7, 14, and 28 | 1 and 2 |
| 5 g straw or burning residuals in 500 mL water **without soil** | straw, black carbon, or ash application | 3, 16, and 22 | 3 |
| 5 g straw or burning residuals in 500 mL water **with 800 g soil** | control, straw, black carbon, or ash application | 1, 3, 5, 7, 10, 16, 22, and 50 | 4, 5 and 6 |
